# Supplementary material for: An Original ELISA-Based Multiplex Method for the Simultaneous Detection of 5 SARS-CoV-2 IgG Antibodies Directed against Different Antigens
Source: J Clin Med. 2020 Nov 21;9(11):3752. doi: 10.3390/jcm9113752 (PMC7700260; doi:10.3390/jcm9113752)
Supplement: Supplementary file 1 [file jcm-09-03752-s001.pdf]

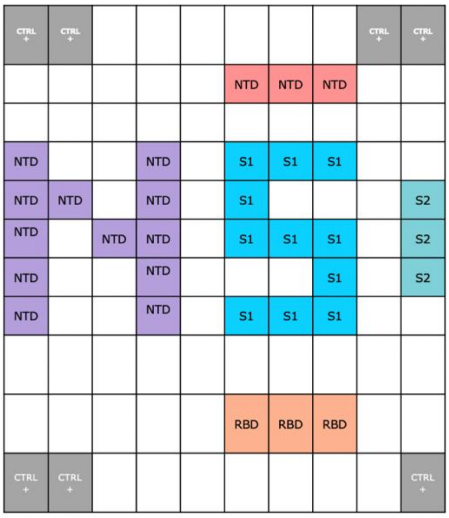

Supplemental Figure 1: Schematic representation of the spiking of the antigens at the bottom of each well. CTRL+: positive control; N: nucleocapsid protein; S1: subunit 1 of the spike protein; RBD: S1 protein receptor binding domain; NTD: N-terminal domain of protein S1; S2: subunit 2 of the spike protein.

Antigen positive	False-positive AU value	Cross-reactive serum
S1	8.24#	Toxoplasmosis IgM
S2	67.9	*Coxiella burnetii* IgM
S2	37.2*	Hepatitis B antigen
S2	16.1*	Hepatitis B antigen
S2	32.6*	Influenza A antibodies
S2	15.9*	IgM cytomegalovirus
S2	24.5*	High level of total IgG
S2	21.1*	Healthy volunteers
S2	20.8*	Healthy volunteers
S2	16.5*	Healthy volunteers
S2	17.3*	Healthy volunteers
RBD	27.1	IgM parvovirus
RBD	16.7*	Healthy volunteers
RBD	16.4*	Rheumatoid factor
NTD	20.3	IgM *Toxoplasma gondii*

Supplemental Table 1: Description of false-positive samples according to the positive antigen, the AU value of the antigen concerned and the potentially cross-reactive antibody. * = not positive when adapted cut-offs are used. # = positive when adapted cut-offs are used.

	Days since symptomes onset	0-6	7-13	14-20	21-28	>28
	n	23	26	24	25	37
N	n positive (≥12 AU)	5	17	24	21	34
	Positivity rate	21.7%	63.0%	100%	84.0%	91.9%
S1	n positive (≥7 AU)	2	8	20	19	32
	Positivity rate	8.7%	29.6%	83.3%	76.0%	86.5%
S2	n positive (≥39 AU)	3	11	21	20	30
	Positivity rate	13.0%	40.7%	87.5%	80.0%	81.1%
RBD	n positive (≥18 AU)	2	5	19	18	31
	Positivity rate	8.7%	18.5%	79.2%	72.0%	83.8%
NTD	n positive (≥16 AU)	0	0	2	2	5
	Positivity rate	0%	0%	8.3%	8.0%	13.5%
≥ 1 antibody	n positive	5	17	24	23	36
	Positivity rate	18.5%	63.0%	100%	92.0%	97.3%

Supplemental Table 2: Evolution of the positivity rates according to the number of days since the onset of symptoms for each antigen (at least 1 positive antigen based on the adapted cut-offs).
